# Activation of CXCR7 exerts an inhibitory effect on adipogenesis through regulation of β-arrestin2/Wnt and AKT signalling

**DOI:** 10.1080/21623945.2025.2490258

**Published:** 2025-04-29

**Authors:** Shiyue Sun, Muhammad Arif Aslam, Eun Bi Ma, Gahui Lee, Hafiz Muhammad Ahmad Javaid, Somy Yoon, Joo Young Huh

**Affiliations:** a College of Pharmacy, Chonnam National University, Gwangju, Republic of Korea; b Department of Anesthesia and Critical Care, The Second Affiliated Hospital of Wenzhou Medical University, Wenzhou, China; c College of Pharmacy, Chung-Ang University, Seoul, Republic of Korea; d Department of Global Innovative Drugs, The Graduate School of Chung-Ang University, Seoul, Republic of Korea

**Keywords:** Adipogenesis, SDF-1, CXCR7, β-arrestin2, Wnt

## Abstract

CXCR7, an alternative receptor for the inflammatory chemokine SDF-1, is involved in cell proliferation and migration. Recent studies have reported that CXCR7 also plays a role in adipose tissue. However, evidence regarding the role of CXCR7 and its ligands in adipocyte differentiation is limited. In this study, we aimed to elucidate changes in CXCR7 expression during adipocyte differentiation and the role of the SDF-1/CXCR7/CXCR4 axis in adipogenesis using recombinant SDF-1, the CXCR7 ligand CCX771, and small interfering RNAs. The results indicated that the levels of SDF-1 and its receptors, CXCR7 and CXCR4, decreased during the early stages of adipogenesis. Treatment with recombinant SDF-1 and CCX771 inhibited adipogenesis and lipid accumulation by inducing β-arrestin2, Wnt expression, and AKT phosphorylation and downregulating C/EBPα, PPARγ, and FABP4 expression. In contrast, knockdown of SDF-1 and CXCR7 in preadipocytes downregulated the β-arrestin2/Wnt and AKT pathway, leading to the induction of adipogenesis. Meanwhile, knockdown of CXCR4 had no significant effect. In mice, basal gene expression levels of SDF-1 and CXCR7 were higher in the stromal vascular fraction compared to mature adipocytes and were significantly upregulated by a high-fat diet. Our results provide new insights into the local role of the SDF-1–CXCR7 axis in adipocytes and offer additional benefits for the prevention of obesity-related metabolic disorders.

## Introduction

Obesity is a rising global epidemic driven by lifestyle changes. The imbalance between food intake and energy expenditure in obesity leads to excess lipid accumulation in adipose tissue, causing dysregulation of adipocyte metabolism and pathophysiological changes, such as type 2 diabetes, hypertension, and cardiovascular disease [1,2]. Adipose tissue expands through two pathways: an increase in the number of adipocytes (hyperplasia) and an increase in adipocyte size (hypertrophy) [3]. Hyperplasia results from an increase in adipocyte numbers, primarily dependent on adipogenesis, where preadipocytes differentiate into mature adipocytes [4]. Adipogenesis is regulated by a complex interplay of transcription factors, including peroxisome proliferator-activated receptor (PPARγ), CCAAT/enhancer-binding proteins (like C/EBPα), and fatty acid-binding protein 4 (FABP4) [5]. Among these, PPARγ and C/EBPα are key regulators in the early differentiation process of adipocytes, while FABP4 is involved in mature adipocyte formation [6]. Moreover, transcription factors are regulated by epigenetic modifiers through Wnt/β-catenin signalling [7]. The canonical Wnt/β-catenin pathway, activated by ligands such as Wnt6, Wnt10a, and Wnt10b, inhibits early adipogenic differentiation [8].

The chemokine system, comprising chemokines and chemokine receptors, directs inflammatory leukocytes into obese adipose tissue and promotes obesity-induced adipose tissue inflammation [9]. Kabir et al. identified chemokine profiles during adipogenesis, revealing that among chemokine receptors, CXCR7 is predominantly expressed in preadipocytes [10]. CXCR7 is an alternative receptor for chemokine stromal cell-derived factor 1 (SDF-1), also known as CXCL12, which plays significant roles in tumour growth, metastasis, and peripheral vascular diseases [11,12]. Previous studies have suggested that CXCR7 induces cell migration and is closely associated with the differentiation of various cells [13–15]. Recent research indicates that CXCR7 is also crucial in adipose tissue [16,17], showing upregulation in obese, Western diet-fed mice, suggesting that adipocyte CXCR7 may be involved in obesity pathology [16]. The high expression of CXCR7 in preadipocytes suggests the development of the SDF-1/CXCR7 axis, but its role in regulating adipogenesis remains unknown.

In this study, we aimed to investigate the role of CXCR7 in adipogenesis compared to CXCR4, another chemokine receptor binding to SDF-1. Recombinant SDF-1 and CCX771, a small molecule with high affinity and selectivity for CXCR7, and siRNAs were used to examine the effect of CXCR4 stimulation on adipocyte differentiation and explore the related mechanistic pathways.

## Results

### CXCR7, CXCR4, and their ligand SDF-1 are reduced during adipogenesis

**Figure 1.**
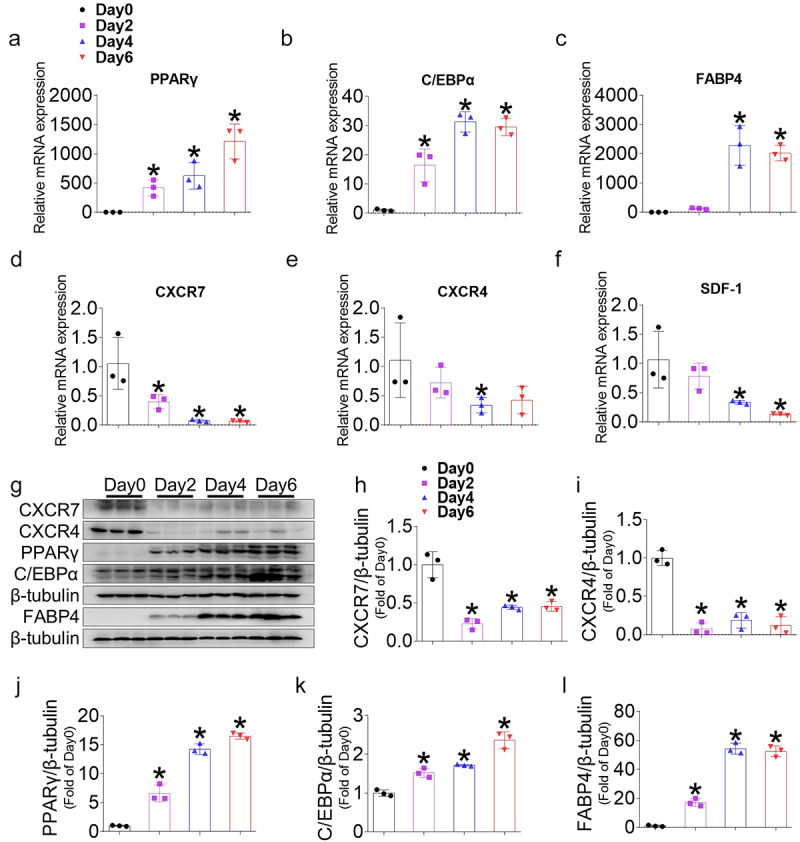
Expression of CXCR7, CXCR4, and SDF-1 during adipogenesis in 3T3-L1 cells. (a-c) Gene expressions of adipogenic markers, PPARγ, C/EBPα, FABP4 and (d-f) the gene expression levels of CXCR7, CXCR4, and SDF-1 were measured using real-time PCR at corresponding time points during adipogenesis. Quantifications were normalized to 18s rRNA for each target gene. (g) Representative western blot images for CXCR7, CXCR4, PPARγ, C/EBPα, and FABP4 in 3T3-L1 cells at day 0, 2, 4, 6 during adipogenesis. (h-l) Quantitative data of CXCR7, CXCR4, PPARγ, C/EBPα, and FABP4 levels normalized to β-tubulin. Values are presented as mean ± standard error of the mean (*n* = 3). *p < 0.05 compared with day 0.

First, we examined the gene expression of CXCR7, CXCR4, and SDF-1, and the protein levels of CXCR7 and CXCR4 during adipocyte differentiation. Preadipocytes successfully differentiated into mature adipocytes by day 6 of differentiation, as demonstrated by the accumulation of lipid droplets in the cytoplasm in over 90% of the cells (Figure S1). In contrast to the upregulation of adipogenic markers PPARγ, C/EBPα, and FABP4 mRNA expression on days 2, 4, and 6 compared to preadipocytes (day 0) (Figure 1(a-c)), the gene expression levels of CXCR7, CXCR4, and SDF-1 were significantly downregulated when cells were treated with the differentiation medium (Figure 1(d-f)). Consistently, the protein levels of CXCR7 and CXCR4 decreased significantly from day 2, while the protein levels of PPARγ, C/EBPα, and FABP4 increased during differentiation (Figure 1(g-l)). Since the PPARγ agonist rosiglitazone was used for adipocyte differentiation, we employed an alternative method using IBMX to induce adipogenesis, avoiding the effect of PPARγ activation. Similar results were obtained, showing that CXCR7, CXCR4, and SDF-1 gene and protein expression levels were significantly downregulated during adipogenesis (Figure S2), indicating that the change was independent of PPARγ activation. These results demonstrate that the expression of CXCR7, CXCR4, and SDF-1 were downregulated during the early stages of adipocyte differentiation and negatively correlates with adipogenic marker expression.

### Deficiency in SDF-1 and CXCR7, but not CXCR4, induces adipocyte differentiation

**Figure 2.**
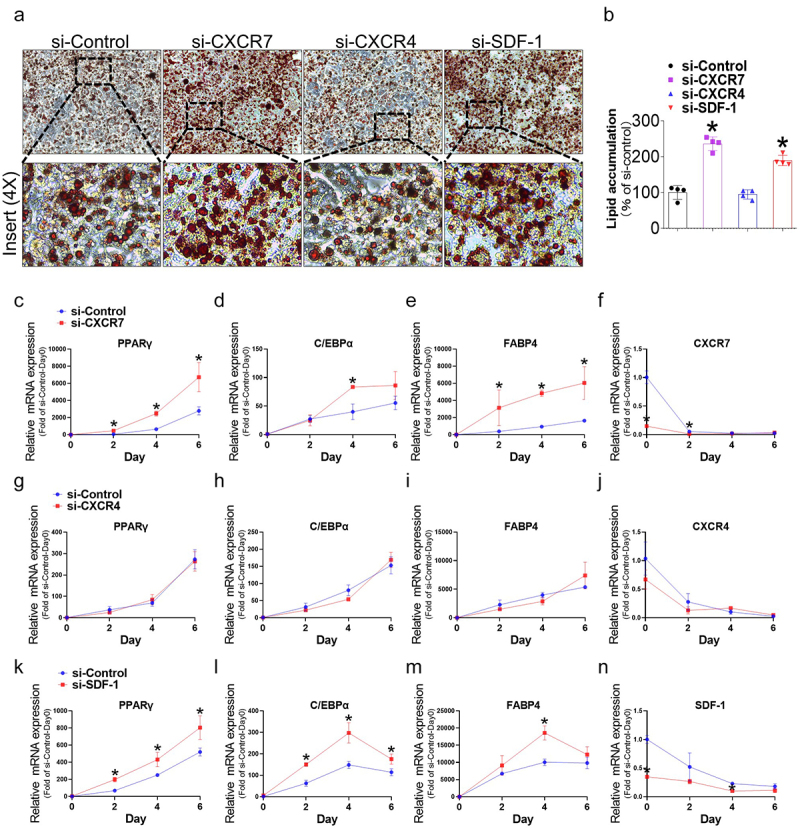
Adipocyte differentiation and gene expression changes of adipogenic markers after CXCR7, CXCR4, and SDF-1 silencing in 3T3-L1 cells. (a) Representative images of oil red O (ORO) staining showing lipid accumulation in differentiated adipocytes. (b) Quantitative data of ORO staining to assess lipid accumulation during adipogenesis. (c-f) Gene expression changes of adipogenic markers after CXCR7 silencing during adipogenesis in 3T3-L1 cells. (g-j) Gene expression changes of adipogenic markers after CXCR4 silencing during adipogenesis in 3T3-L1 cells. (k-n) Gene expression changes of adipocyte markers after SDF-1 silencing during adipogenesis in 3T3-L1 cells. Quantifications were normalized to 18s rRNA for each target gene. Values are presented as mean ± standard error of the mean (*n* = 3). *p < 0.05 compared with si-control at each time point.

**Figure 3.**
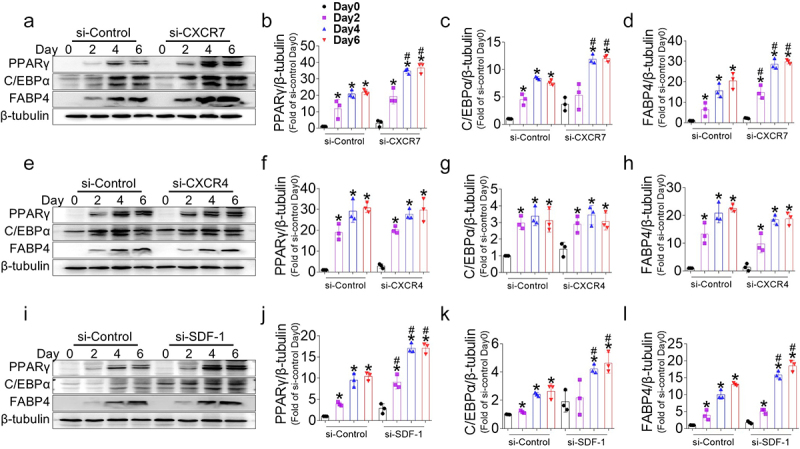
Protein expression changes of adipogenic markers after CXCR7, CXCR4, and SDF-1 silencing in 3T3-L1 cells. (a) Representative western blot images showing the protein levels of adipogenic markers after CXCR7 silencing in 3T3-L1 cells. (b-d) Quantitative data of PPARγ, C/EBPα, and FABP4 protein levels normalized to β-tubulin after CXCR7 silencing. (e) Representative western blot images showing the protein levels of adipogenic markers after CXCR4 silencing in 3T3-L1 cells. (F-H) quantitative data of PPARγ, C/EBPα, and FABP4 protein levels normalized to β-tubulin after CXCR4 silencing. (i) Representative western blot images showing the protein levels of adipogenic markers after SDF-1 silencing in 3T3-L1 cells. (j-l) Quantitative data of PPARγ, C/EBPα, and FABP4 protein levels normalized to β-tubulin after SDF-1 silencing. Values are presented as mean ± standard error of the mean (*n* = 3). *p < 0.05 compared with day 0. #*p* < 0.05 compared with si-control at each time point.

The downregulation of CXCR7, CXCR4, and SDF-1 in early differentiating cells suggests that the inhibition of these factors may be required for the induction of adipogenesis. To evaluate their causal role in adipocyte differentiation, preadipocytes were treated with siRNAs to knock down CXCR7, CXCR4, and SDF-1 on day 0 (Figure 2(f,j,n) and Figure S3). Oil Red O staining on day 6 post-differentiation showed a significant increase in mature adipocytes in CXCR7 or SDF-1 knockdown cells compared to the control, resulting in increased lipid accumulation (Figure 2(a-b)). In contrast, there was no difference in adipocyte differentiation between CXCR4 knockdown and control cells, as evidenced by similar levels of lipid accumulation. We then assessed the effect of CXCR7, CXCR4, and SDF-1 deficiency on PPARγ, C/EBPα, and FABP4 gene and protein expression. Continuous monitoring at days 0, 2, 4, and 6 of differentiation revealed that CXCR7 deficiency significantly increased the gene (Figure 2(c-e)) and protein (Figure 3(a-d)) expression of PPARγ, C/EBPα, and FABP4 during adipogenesis. Interestingly, SDF-1 knockdown yielded similar results to CXCR7 knockdown (Figures 2(k-m), 3(i-l)), implying an autocrine role for SDF-1 on the receptor. In contrast, silencing CXCR4 had no significant effect on PPARγ, C/EBPα, and FABP4 gene and protein expression (Figures 2(g-i), 3(e-h)). These findings indicate that suppression of CXCR7 or SDF-1 induces adipogenesis thorough induction of PPARγ, C/EBPα, and FABP4, whereas suppression of CXCR4 has no significant effect.

### Recombinant SDF-1 treatment inhibits adipocyte differentiation

**Figure 4.**
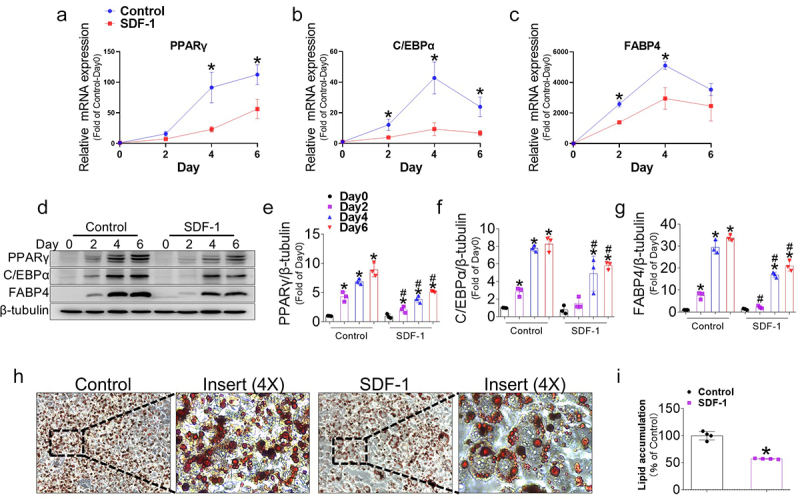
Recombinant SDF-1 treatment inhibits adipogenesis in 3T3-L1 cells. (a-c) Gene expressions of adipogenic markers in 3T3-L1 cells treated with or without SDF-1 were measured using real-time PCR at different time points during adipogenesis. Quantifications were normalized to 18s rRNA for each target gene. (d) Representative western blot images showing the protein levels of adipogenic markers in 3T3-L1 cells treated with or without SDF-1 during adipogenesis at different time points during adipogenesis. (e-g) Quantitative data of PPARγ, C/EBPα, and FABP4 protein levels normalized to β-tubulin. (H) Representative images of oil red O (ORO) staining at day 6, showing lipid accumulation in differentiated adipocytes treated with or without SDF-1. (i) Quantitative data of ORO staining in 3T3-L1 cells treated with or without SDF-1. Values are presented as mean ± standard error of the mean (*n* = 3). (a-c, i) *p < 0.05 compared with control at each time point. (D-G) *p < 0.05 compared with day 0. #*p* < 0.05 compared with control at each time point.

To further validate the role of the CXCR7 ligand in adipocyte differentiation, adipocytes were treated with 100 ng/mL recombinant SDF-1 during adipogenesis. As a result, SDF-1 treatment downregulated the gene expressions of PPARγ, C/EBPα, and FABP4 at days 2, 4, and 6 of the differentiation period (Figure 4(a-c)). Consistently, the protein expression levels of PPARγ, C/EBPα, and FABP4 in SDF-1-treated cells were also significantly decreased at days 2, 4, and 6, compared to controls on the same days (Figure 4d-g). Furthermore, Oil Red O staining confirmed that SDF-1 treatment decreased lipid accumulation on day 6 (Figure 4(h, i)). These findings suggest that SDF-1 inhibits adipogenesis in 3T3-L1 cells.

### Treatment of CXCR7 ligand CCX771 inhibits adipocyte differentiation

**Figure 5.**
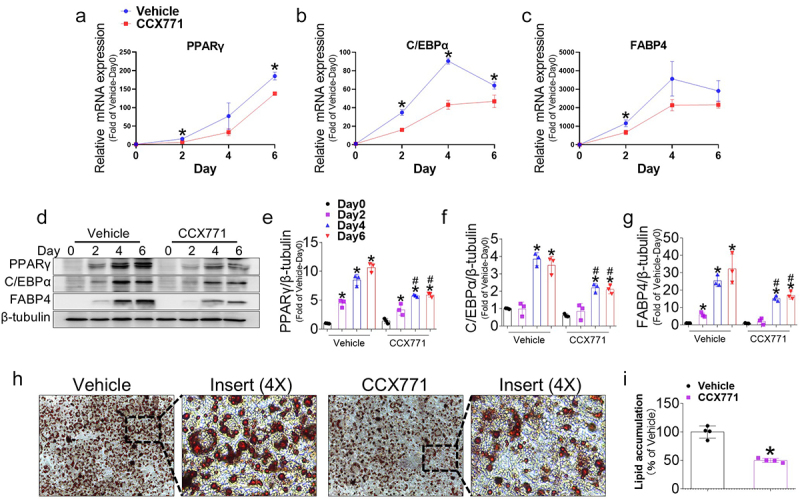
CXCR7 ligand CCX771 inhibits adipogenesis in 3T3-L1 cells. (a-c) Gene expressions of adipogenic markers in 3T3-L1 cells treated with or without CCX771 were measured using real-time PCR at different time points during adipogenesis. Quantifications were normalized to 18s rRNA for each target gene. (d) Representative western blot images showing the protein levels of adipogenic markers in 3T3-L1 cells treated with or without CCX771 at different time points during adipogenesis. (e-g) Quantitative data of PPARγ, C/EBPα, and FABP4 protein levels normalized to β-tubulin. (h) Representative images of oil red O (ORO) staining at day 6, showing lipid accumulation in differentiated adipocytes treated with or without CCX771. (i) Quantitative data of ORO staining in 3T3-L1 cells treated with or without CCX771 at day 6. Values are presented as mean ± standard error of the mean (*n* = 3). (a-c, i) *p < 0.05 compared with vehicle at each time point. (d-g) *p < 0.05 compared with day 0. #*p* < 0.05 compared with vehicle at each time point.

SDF-1 is the ligand for both CXCR7 and CXCR4, and since SDF-1/CXCR4 can also play an important role in cell differentiation [18,19], we used CCX771, a synthetic ligand of CXCR7, to eliminate the influence of CXCR4 and specifically activate CXCR7. Accordingly, the effect of CCX771 was assessed by changes in the gene expression and protein levels of PPARγ, C/EBPα, and FABP4 during adipogenesis. Consistent with the SDF-1 treatment results, CCX771 treatment downregulated the expression of these transcription factors at days 2, 4, and 6 of the differentiation period (Figure 5(a-c)), and protein expression was also decreased compared with the controls (Figure 5(d-g)). Oil Red O staining revealed that CCX771 treatment reduced lipid accumulation on day 6 (Figure 5(h,i)). Taken together, these results indicate that CXCR7 activation inhibits adipogenesis in 3T3-L1 cells.

### Deficiency in CXCR7 and SDF-1 results in suppression of the β-Arrestin2/Wnt and AKT signaling pathway

**Figure 6.**
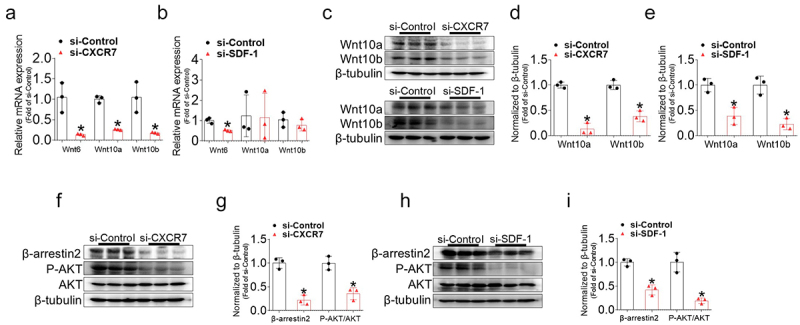
CXCR7 or SDF-1 silencing inhibits β-arrestin2/Wnt and AKT signalling pathway in 3T3-L1 cells. (a-b) Gene expression levels of Wnt6, Wnt10a, and Wnt10b in 3T3-L1 cells with or without CXCR7 or SDF-1 silencing were measured using real-time PCR at day 6 of differentiation. Quantifications were normalized to 18s rRNA levels. (c) Representative western blot images of Wnt10a and Wnt10b in 3T3-L1 cells with or without CXCR7 or SDF-1 silencing. (d-e) Quantitative data of Wnt10a and Wnt10b protein levels normalized to β-tubulin. (f, h) Representative western blot images of β-arrestin, phosphorylated AKT (P-AKT), and total AKT (AKT) in 3T3-L1 cells with or without CXCR7 or SDF-1 silencing. (g, i) Quantitative data of β-arrestin normalized to β-tubulin and the ratio of P-AKT to AKT levels in 3T3-L1 cells with or without CXCR7 or SDF-1 silencing. Values are presented as mean ± standard error of the mean (*n* = 3). *p < 0.05 compared with si-control.

Next, we examined the mechanisms underlying the effects of SDF-1 and the CXCR7 axis on adipocyte differentiation. The Wnt/β-catenin pathway functions as a negative regulator of adipogenesis by inhibiting the induction of PPARγ and C/EBPα during the early stages of adipogenic differentiation [7,8]. While we confirmed that the gene and protein expression of various Wnt ligands decreased during early stages of adipogenesis (Figure S4A-D), the knockdown of CXCR7 and SDF-1 further inhibited their expression (Figure 6(a-b)). Specifically, the gene expression of Wnt6, 10a, and 10b was suppressed at day 2 of differentiation when CXCR7 and SDF-1 were silenced. In addition, the protein expression of Wnt10a and Wnt10b significantly decreased at day 2 of differentiation in CXCR7- or SDF-1-deficient adipocytes (Figure 6(c-e)).

Recent reports have reported that SDF-1-stimulated CXCR7 mediates β-arrestin2 recruitment via a molecular network distinct from that of CXCR4 [20]. β-arrestin2 is an essential component of Wnt/β-catenin signalling, and plays a critical role in adipocyte differentiation [21,22]. Our results also confirmed that the protein expression of β-arrestin2 decreases during adipogenesis (Figure S4E). Based on this, the impact of CXCR7 and SDF-1 deficiency on β-arrestin2 expression was examined. Knockdown of CXCR7 and SDF-1 resulted in a significant reduction in β-arrestin2 protein expression. Consistent with the downstream mechanism of CXCR7/β-arrestin2 axis in gastric cancer [23], the decrease in β-arrestin2 led to reduced AKT phosphorylation compared to the nonsense control (Figure 6(f-i)).

### SDF-1 and CCX771 treatment enhances the expression of the β-Arrestin2/Wnt and AKT signaling pathway

**Figure 7.**
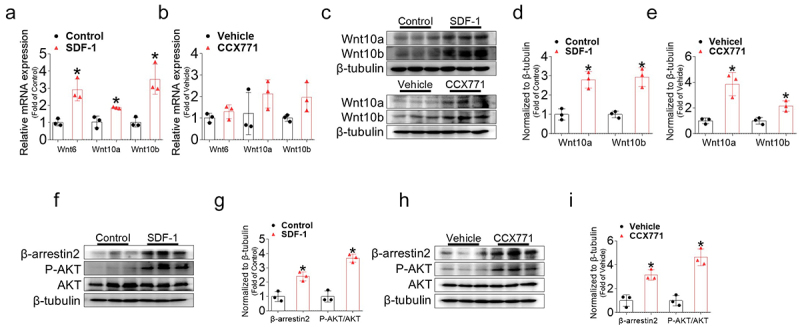
SDF-1 or CCX771 treatment activates β-arrestin2/Wnt and AKT signalling pathways in 3T3-L1 cells. (a-b) Gene expression levels of Wnt6, Wnt10a, and Wnt10b in 3T3-L1 cells treated with or without SDF-1 or CCX771 were measured using real-time PCR at day 6 of differentiation. Quantifications were normalized to 18s rRNA levels. (c) Representative western blot images of Wnt10a and Wnt10b in 3T3-L1 cells treated with or without SDF-1 or CCX771. (d-e) Quantitative data of Wnt10a and Wnt10b protein levels normalized to β-tubulin. (f, h) Representative western blot images of β-arrestin, phosphorylated AKT (P-AKT), and total AKT (AKT) in 3T3-L1 cells treated with or without SDF-1 or CCX771. (g, i) Quantitative data of β-arrestin normalized to β-tubulin and the ratio of P-AKT to AKT levels in 3T3-L1 cells treated with or without SDF-1 or CCX771. Values are presented as mean ± standard error of the mean (*n* = 3). *p < 0.05 compared with control.

To validate, 3T3-L1 preadipocytes were treated with recombinant SDF-1 and CCX771 from day 0. The expression levels of Wnt6, Wnt10a, and Wnt10b genes were significantly elevated following SDF-1 treatment, with a similar increasing trend observed after CCX771 treatment compared to the controls (Figure 7(a,b)). Additionally, Wnt10a and Wnt10b protein levels were notably upregulated by both SDF-1 and CCX771 treatments (Figure 7(c-e)). Furthermore, both β-arrestin2 levels and AKT phosphorylation were significantly increased by SDF-1 or CCX771 treatment during adipose differentiation (Figure 7(f-i)). These results imply that SDF-1/CXCR7 can inhibit adipogenesis by enhancing the expression of the β-arrestin2 and downstream Wnt and AKT signalling pathway.

### SDF-1 and CXCR7 gene expressions are upregulated in adipose tissue of high-fat diet-fed mice and are higher in the stromal vascular fraction compared to adipocytes

**Figure 8.**
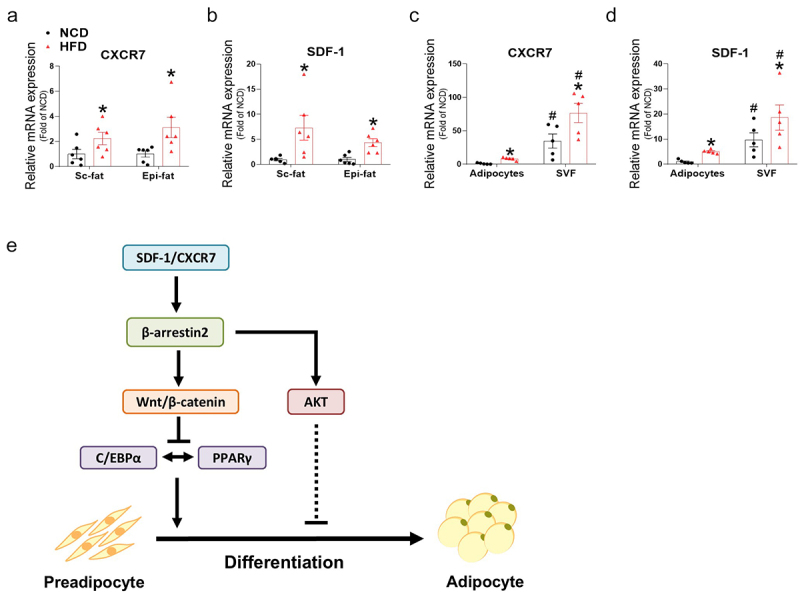
SDF-1 and CXCR7 gene expressions in adipose tissue of high-fat diet-fed mice and isolated stromal vascular fraction and mature adipocytes. (a-b) Gene expression levels of CXCR7 and SDF-1 in subcutaneous fat (sc-fat) and epididymal fat (epi-fat) in normal chow diet (NCD) and high-fat diet (HFD)-fed mice. Mice were fed HFD for 12 weeks. (c-d) Gene expression levels of CXCR7 and SDF-1 in stromal vascular fraction (SVF) and adipocytes isolated from adipose tissues of NCD and HFD mice. (e) Schematic diagram of the effect of SDF-1/CXCR7 on adipogenesis. Activation of CXCR7 by SDF-1 leads to upregulation of β-arrestin2 and wnt, which inhibits PPARγ and C/EBPα. SDF-1/CXCR7 activation also leads to induction of β-arrestin2/AKT axis, in which the mechanisms on adipogenesis inhibition remains to be investigated. Values are presented as mean ± standard error of the mean (*n* = 5). *p < 0.05 compared with NCD, #*p* < 0.05 compared with respective groups in adipocyte fraction.

To investigate the role of SDF-1/CXCR7 *in vivo*, we measured the gene expression of SDF-1 and CXCR7 in NCD- and HFD-fed mice and further analysed their regulation in the stromal vascular fraction (SVF) and mature adipocytes. Previous studies have suggested that obesity and type 2 diabetes are associated with increased progenitor cell senescence, reduced adipogenesis, and hypertrophic expansion, which contribute to inflammation and insulin resistance [24,25]. To investigate the relationship between CXCR7, SDF-1, and obesity *in vivo*, subcutaneous and epididymal adipose tissues were collected from NCD- and HFD-fed mice. Gene expression analysis revealed that in both adipose tissue types, CXCR7 and SDF-1 levels were significantly elevated in HFD-fed mice compared to NCD-fed mice (Figure 8(a-b)). Furthermore, basal mRNA levels of CXCR7 and SDF-1 were higher in the SVF compared to mature adipocytes (Figure 8(c-d)), suggesting a predominant role for CXCR7 and SDF-1 in the SVF, including preadipocytes. In both the SVF and adipocyte fractions, CXCR7 and SDF-1 mRNA levels were upregulated by HFD, with a much greater magnitude observed in the SVF.

## Discussion

The chemokine system plays important roles in the development of obesity and insulin resistance. Therefore, identifying specific chemokine-receptor axes regulating adipocyte metabolism could allow us to find therapeutic targets to manipulate these diseases. Previous report showed that CXCR7 expression was specific for preadipocytes, indicating the development of a SDF-1/CXCR7 axis in preadipocytes [10]. However, studies on the role of the SDF-1/CXCR7 during adipogenesis are lacking. This study, for the first time, reports that CXCR7 ligands exhibit inhibitory effects on adipogenesis through the regulation of β-arrestin2 and downstream Wnt and AKT signalling (Figure 8(e)). Key findings include: (1) The expression of CXCR7, CXCR4, and SDF-1 decreased in the early stages of adipogenesis. (2) Knockdown of SDF-1 and CXCR7, but not CXCR4, further induced adipogenesis via Wnt and β-arrestin2 signalling. (3) SDF-1 and CCX771, ligands of CXCR7, inhibit adipocyte differentiation by inducing Wnt and β-arrestin2 expression. (4) SDF-1 and CXCR7 gene expressions were upregulated in adipose tissue of HFD mice and were higher in the SVF compared to adipocytes.

Recent study has indicated that CXCR7 and its ligand SDF-1 are closely related to obesity and insulin resistance. CXCR7 expression in adipose tissue macrophages (ATMs) is upregulated in obesity, and blocking CXCR7-mediated ATMs chemotaxis ameliorated insulin resistance and inflammation, suggesting a role of CXCR7 in pathogenesis during obesity [16]. In addition, SDF-1 is reported to be an autocrine insulin-desensitizing factor in adipocytes that induces serine phosphorylation and degradation of the IRS-1 protein, attenuating insulin-mediated Akt phosphorylation and glucose uptake [26]. In this study, we demonstrate using 3T3-L1 cells that SDF-1/CXCR7 activation suppresses adipogenesis. To bridge the gap between *in vitro* and *in vivo* findings, we also show that CXCR7 and SDF-1 levels are elevated in the adipose tissue of HFD-fed mice, particularly in the SVF. Since impaired adipogenesis and hypertrophic expansion are known to contribute to obesity-induced adipose tissue dysfunction [24,25], our findings suggest that an elevated SDF-1/CXCR7 axis may contribute to this dysfunction through two mechanisms: activation of inflammation and limitation of adipogenic differentiation capacity. In contrast to our findings, which showed CXCR7 deficiency induced adipogenesis and increased lipid accumulation in mature adipocytes, another study reported that adipocyte-specific CXCR7 knockout reduced adipose tissue lipid levels in hyperlipidemic Apoe^−/−^ mice [17]. Consistent with our findings, PPARγ levels were elevated in adipocytes with CXCR7 deficiency, indicating a connection between CXCR7 and PPARγ, although its role may differ between preadipocytes and mature adipocytes.

CXCR4 is also a receptor for SDF-1, and both CXCR7 and CXCR4 can mediate SDF-1-induced chemotaxis [27]. However, our results show that only CXCR7 is involved in regulation of adipogenesis. Of note, the downstream mechanisms are known to be different among the two receptors. CXCR4 induces Gαi activation and recruits β-arrestin2 through C-terminal phosphorylation by both GRK2 and GRK5, whereas ligand binding to CXCR7 does not result in the activation of typical signalling pathways via Gα subunits but instead activates GRK2 via βγ subunits and receptor phosphorylation with subsequent β-arrestin2 recruitment [20]. A study showed that CXCR4 deficiency in adipocytes exacerbates obesity and compromises the thermoregulatory responses of brown adipose tissue in a mouse model of diet-induced obesity [28]. Since CXCR4 knockdown had no significant influence on adipogenesis in our study, the effect of CXCR4 on adipose tissue may involve mechanisms other than adipogenesis.

Wnt signalling promotes β-catenin stabilization and nuclear translocation, which subsequently inhibits PPARγ and C/EBPα during the early stages of adipogenesis [8,29]. Our previous study also demonstrates that Wnt activation between days 0 and 2 of differentiation plays an important role in regulating adipogenesis [30]. Since the protein expression of SDF-1, CXCR4, and CXCR7 was also markedly reduced by day 2, we selected this time point to examine their effects on the signalling pathways. As a result, knockdown of CXCR7 or SDF-1 reduced Wnt ligand expression, whereas treatment with SDF-1 or CCX771 restored Wnt ligand expression in 3T3-L1 cells during adipogenesis. Previous studies have reported that CCX771 and SDF-1 can stimulate β-arrestin2 recruitment to CXCR7, activating downstream signalling pathways [20,31]. We found that β-arrestin2 expression correlates with Wnt expression, consistent with its role in adipogenesis [21,22]. CCX771 and SDF-1 axis also induces β-arrestin2 and AKT phosphorylation, similar to CXCR7-induced AKT activation in cancer [23]. Most studies on the role of AKT in adipogenesis have focused on its insulin-induced acute effects in promoting differentiation [32]. In contrast, we show that AKT phosphorylation diminishes as cells mature and that activation of the β-arrestin2/AKT pathway inhibits adipogenesis. This highlights the need for further investigation into how AKT interacts with the SDF-1/CXCR7 axis to inhibit adipogenesis. Since adipogenesis is a highly dynamic process involving the interaction of multiple signalling pathways, it is also necessary to explore the involvement of other pathways, such as mTOR or MAPK. Based on the known mechanistic roles of CXCR7 in cancer [23,33], it is plausible that the SDF-1/CXCR7 axis may regulate additional pathways during adipogenesis.

Studying adipogenesis *in vivo* is challenging due to the presence of multiple cell types within adipose tissue and the metabolic and hormonal complexity of the microenvironment. In this context, our *in vitro* studies using siRNAs and pharmacological treatments have the advantage of selectively targeting SDF-1/CXCR7 in adipogenesis, providing evidence of a causal effect that is difficult to capture *in vivo*. However, our study using 3T3-L1 cells has limitations, as the results may not fully reflect the native adipose tissue environment. While we demonstrated SDF-1 and CXCR7 expression in mice, further experiments employing lineage tracing methods are necessary to clarify their roles *in vivo*.

In conclusion, we found that CXCR7, CXCR4, and SDF-1 levels are reduced in the early stages of adipocyte differentiation and that silencing CXCR7 and SDF-1 enhances adipogenesis through the regulation of β-arrestin2/Wnt and AKT signalling. Moreover, we demonstrated that the ligands of CXCR7 exert inhibitory effects on adipogenesis. In addition to the role of SDF-1/CXCR7 as a chemokine, our data provide evidence of its ability to limit the expansion of adipose tissue, which further highlights its therapeutic potential in obesity-related metabolic disorders.

## Materials and methods

### Cell culture and treatment

3T3-L1 mouse preadipocytes were purchased from ATCC (Manassas, VA, USA). Cells were seeded in 100-mm culture plates for growth, 6-well plates for western blot and gene expression, and 96-well plates for Oil Red O staining and maintained in Dulbecco’s modified Eagle’s medium (DMEM) supplemented with 10% foetal bovine serum (FBS, HyClone; Australia) and 1% penicillin/streptomycin (Carlsbad, CA, USA), incubated at 37°C in 5% CO_2_. Preadipocytes were differentiated as described in our previous study [34]. Briefly, 3T3-L1 cells were treated with differentiation media containing insulin, dexamethasone, isobutylxanthine, and rosiglitazone after reaching 100% confluency. To examine the role of CXCR7, CXCR4, and SDF-1 in adipogenesis, small interfering RNA (siRNAs) for CXCR7, CXCR4, and SDF-1 were used when the cells reached 70–80% confluency. Moreover, 3T3-L1 preadipocytes were also treated with 100 ng/mL recombinant SDF-1 (HY-P70518) or 5 μM CCX771 (ChemoCentryx (CA, USA)) on day 0 and 2 to examine the effect of CXCR7, CXCR4, and SDF-1 on adipocyte differentiation.

### Animal study

The animal study was conducted as previously described [35]. Male 5-week-old C57BL/6 mice (Samtako Bio, South Korea) were fed either a normal chow diet (NCD) or a high-fat diet (HFD; Research Diets, New Brunswick, NJ) for 12 weeks. Subcutaneous and epididymal adipose tissues were collected and stored at −80°C at the time of sacrifice. For the fractionation of SVF and adipocytes, epididymal adipose tissue from NCD- and HFD-fed mice was digested with 1 mg/mL collagenase I (Gibco, Waltham, MA, USA) in a buffer containing 5 M NaCl, 2 M KCl, 1 M CaCl2, 1.5% BSA, 5 mm glucose, and 1 M HEPES for 30 minutes in a water bath at 37°C. The digested tissue was filtered through a 100 μm mesh (Corning, NY, USA) and centrifuged at 2,000 rpm for 3 minutes. The isolated SVF pellet and supernatant adipocytes were stored at −80°C for gene expression analysis. All animal experiments were approved by the Institutional Animal Care and Use Committee of Chonnam National University, and the study was designed and reported following the ARRIVE guidelines.

### Gene expression analysis

As previously reported [36], total RNA was isolated from 3T3-L1 cells using TRI Reagent (MRC, Cincinnati, OH, USA). cDNA synthesis was performed using TOPscript™ RT DryMIX (Enzynomics, Daejeon, South Korea). mRNA expression levels were quantified by RT-PCR on a Rotor-Gene Q (QIAGEN, Hilden, Germany) in a 20 μL reaction volume, which included primer pairs, cDNA templates, and TOPreal SYBR Green PCR Kit (Enzynomics). Expression levels of target genes were normalized to 18S rRNA.

### Western blotting

In brief, cell samples were kept on ice and lysed using RIPA buffer (Thermo Scientific, Rockford, IL, USA). Protein concentration was measured with a BCA kit (Thermo Scientific, Rockford, IL, USA), and equal amounts of protein were separated by SDS-PAGE and transferred onto PVDF membranes. As described previously [37], the membranes were blocked with 5% skim milk at room temperature for 1 hour, followed by an overnight incubation at 4°C with primary antibodies. After incubation, the membranes were washed with Tris-buffered saline containing Tween-20 (TBST) and incubated with secondary antibodies for 1 hour at room temperature. Protein bands were visualized using the LAS-4000 system (Fuji Photo Film, Tokyo, Japan). PPARγ (#2435, 1:2000), C/EBPα (#8178, 1:2000), FABP4 (#3544, 1:2000), phospho-AKT (#9271, 1:2000), total-AKT (#9272, 1:2000), and β-tubulin (#2146, 1:2000) antibodies were purchased from Cell Signaling Technology (Danvers, MA, USA). CXCR4 (sc -53,534, 1:1000), β-arrestin2 (sc -13,140, 1:1000), mouse anti-rabbit IgG-HRP (#sc-2357, 1:2000), and m-IgGƙ BP-HRP (#sc -516,102, 1:2000) were procured from Santa Cruz Biotechnology Inc. (Dallas, TX, USA), and CXCR7 (20423–1-AP, 1:1000) antibody was from Proteintech.

### siRNA transfection

To knockdown CXCR7, CXCR4, and SDF-1 expression in 3T3-L1 cells, siRNAs against mouse CXCR7, CXCR4, SDF-1, or Silencer Select Negative Control (Sigma) were transfected into 3T3-L1 cells using Lipofectamine 2000 (Thermo Fisher Scientific). The siRNAs against CXCR7, CXCR4, and SDF-1 sequences were as follows: CXCR7 siRNA sense: CUCACUAUGGAUGUACCUU, antisense: AAGGUACAUCCAUAGUGAG. CXCR4 siRNA sense: CUCUAGUUUUUGUGAGGUU, antisense: AACCUCACAAAAACUAGAG. SDF-1 siRNA sense: CUGCAUCAGUGACGGUAAA, antisense: UUUACCGUCACUGAUGCAG.

### Oil red O staining

Oil Red O (ORO) staining was performed 6 days after the differentiation of 3T3-L1 cells. Firstly, the cells were fixed with 10% formalin for 30 min and briefly washed with distiled water, then incubated with 0.3% ORO working solution (Sigma-Aldrich, St. Louis, MO, United States) at room temperature for 2 h and again washed. Images were captured using a microscope. For quantification, 200 µl 100% isopropanol was added to each well, and the absorbance was measured at 490 nm.

### Statistical analysis

Mean values were derived from three independent experiments. All data analyses were performed using GraphPad Prism software (version 5.0) with a one-way ANOVA, followed by Fisher’s Protected Least Significant Difference (PLSD) post-hoc test. Statistical significance was set at *p* < 0.05.

## Supplementary Material

Supplemental Material

## Data Availability

The data that support the findings of this study are available in figshare at https://doi.org/10.6084/m9.figshare.26827288.v2
