# Supplementary material for: Activation of CXCR7 exerts an inhibitory effect on adipogenesis through regulation of β-arrestin2/Wnt and AKT signalling
Source: Adipocyte. 2025 Apr 29;14(1):2490258. doi: 10.1080/21623945.2025.2490258 (PMC12045560; doi:10.1080/21623945.2025.2490258)
Supplement: Supplemental Material [file KADI_A_2490258_SM5379.docx]

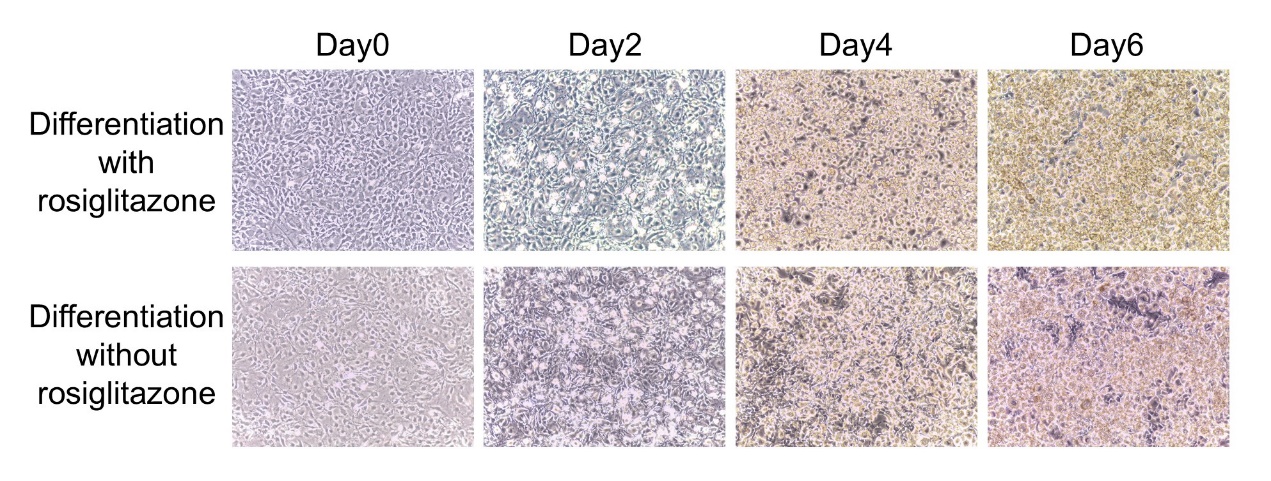


**Figure S1. The lipid level in 3T3-L cells during adipogenesis.** Representative images showing the change in lipid levels on day 0, 2, 4, and 6 in 3T3-L cells by two differentiation media.


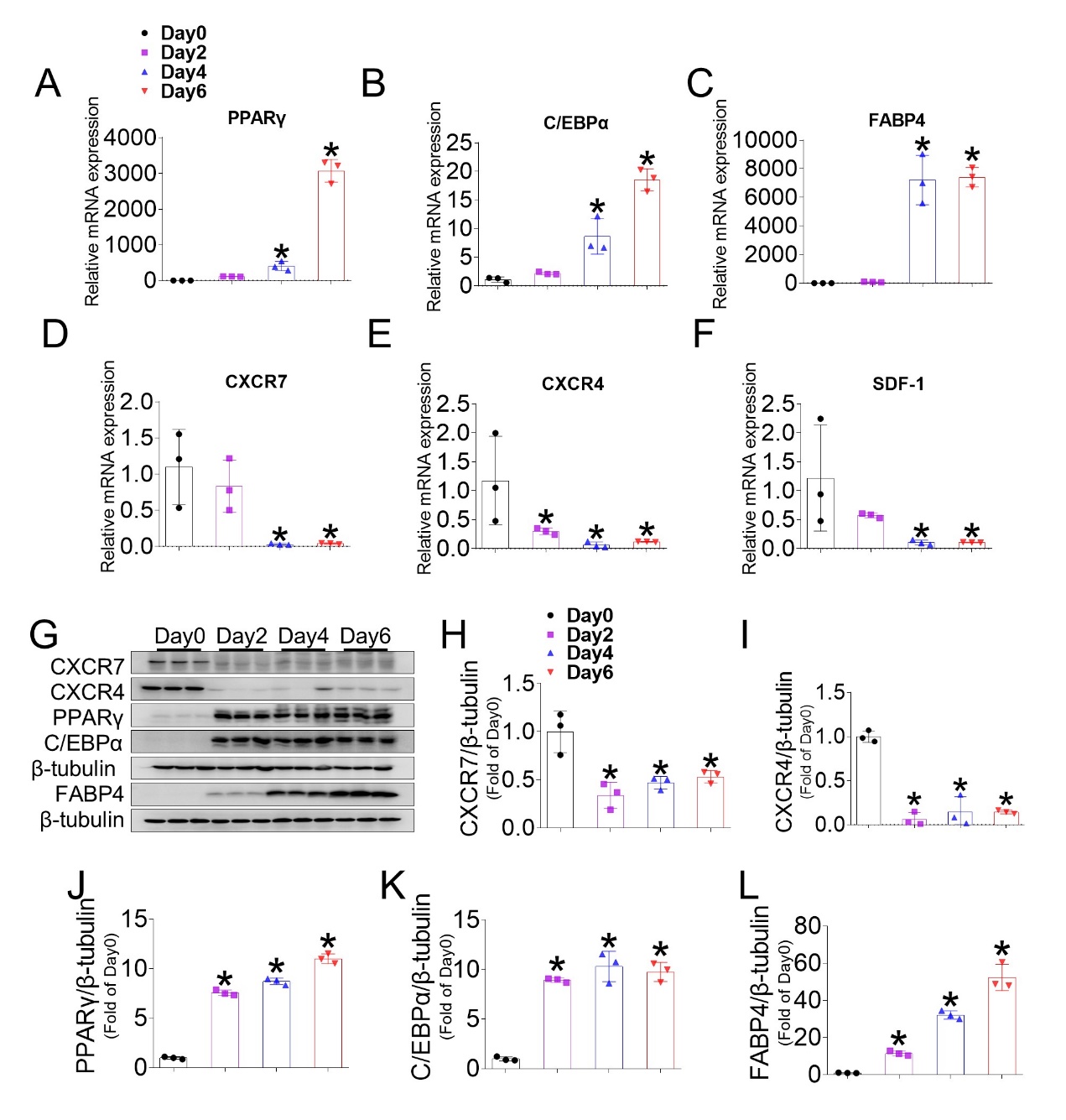


**Figure S2. Expression of CXCR7, CXCR4, and SDF-1 during adipogenesis in 3T3-L1 cells treated without rosiglitazone.** (A-C) Gene expressions of adipogenic markers, PPARγ, C/EBPα, FABP4 and (D-F) the gene expression levels of CXCR7, CXCR4, and SDF-1 were measured using real-time PCR at corresponding time points during adipogenesis. Quantifications were normalized to 18s rRNA for each target gene. (G) Representative western blot images for CXCR7, CXCR4, PPARγ, C/EBPα, and FABP4 in 3T3-L1 cells at day 0, 2, 4, 6 during adipogenesis. (H-L) Quantitative data of CXCR7, CXCR4, PPARγ, C/EBPα, and FABP4 levels normalized to β-tubulin. Values are presented as mean ± standard error of the mean (n = 3). * *p* < 0.05 compared with Day 0.


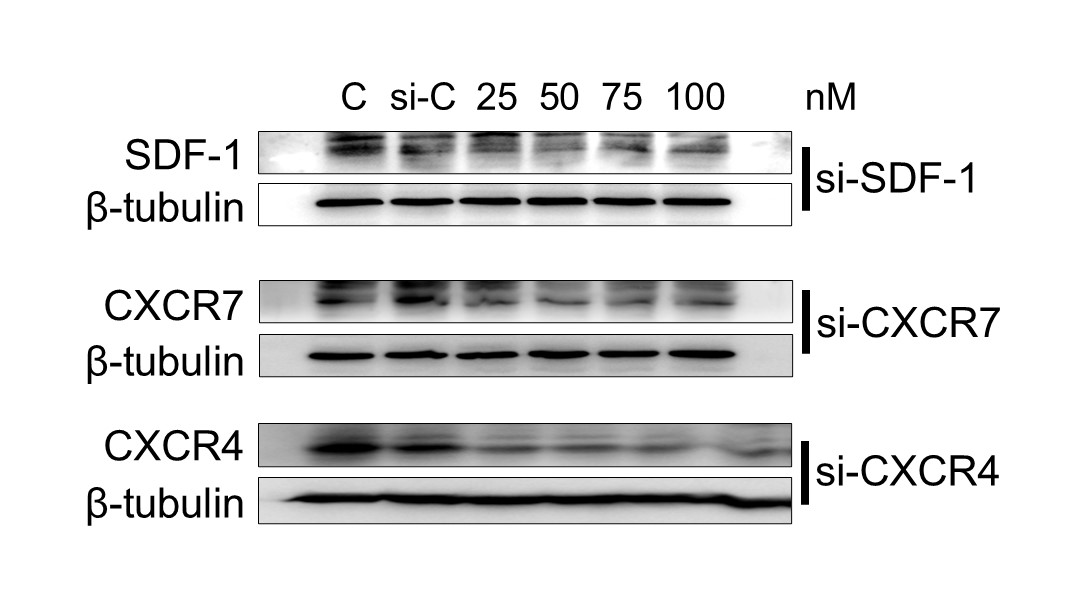


**Figure S3. Expression of SDF-1, CXCR7, and CXCR4 in response to siRNA treatment at different concentrations in 3T3-L1 cells.** Representative western blot images showing the protein levels of SDF-1, CXCR7, and CXCR4 in 3T3-L1 cells treated with si-SDF-1, si-CXCR7, si-CXCR4 at the concentrations of 25, 50, 75 and 100 nM and si-Control (si-C).


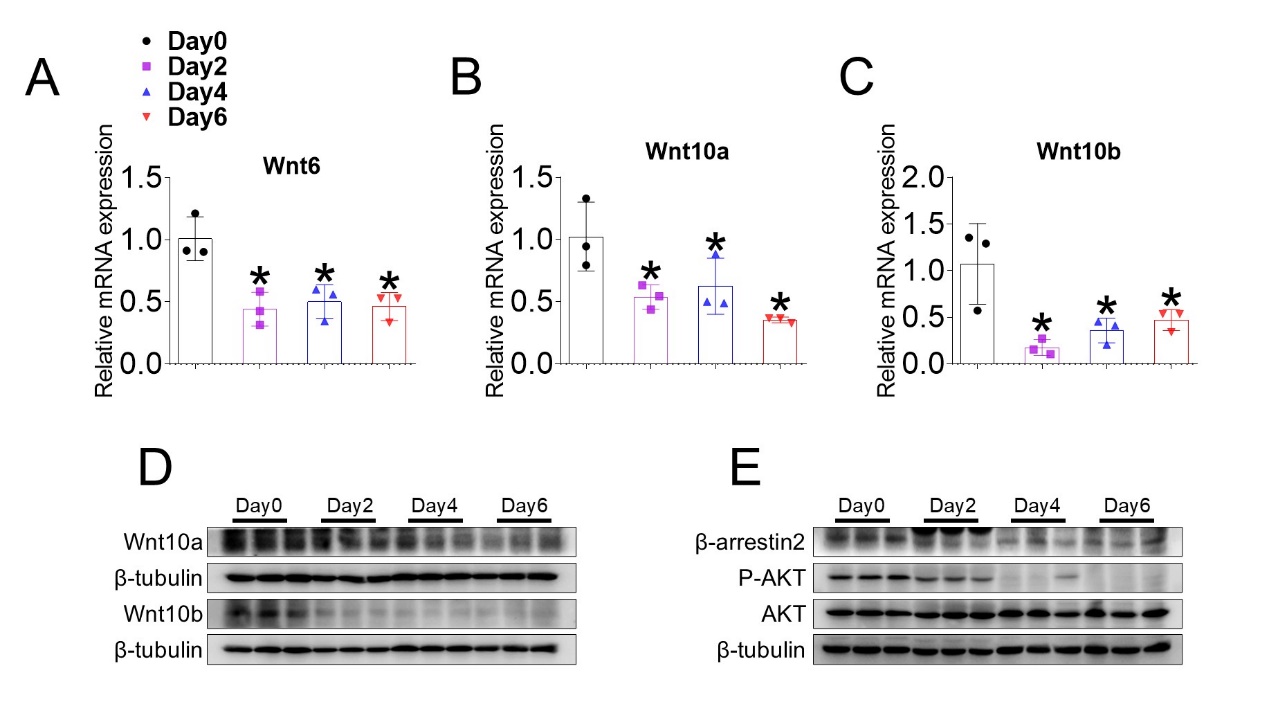


**Figure S4. Expression of Wnt and β-arrestin2/AKT signaling pathways during adipogenesis in 3T3-L1 cells.** (A-C) The gene expression of Wnt6, Wnt10a, and Wnt10b in 3T3-L1 cells at day 0, 2, 4, 6 were measured by using real-time PCR and quantifications were normalized to 18s rRNA levels. (D) Representative western blot images showing the protein levels of Wnt10a, Wnt10b in 3T3-L1 cells during adipogenesis. (E) Representative western blot images showing the protein levels of β-arrestin, p-AKT, AKT in 3T3-L1 cells during adipogenesis. Values are presented as mean ± standard error of the mean (n = 3). * *p*< 0.05 compared with Day 0.
